# Impaired Cognitive Function and Hippocampal Changes Following Chronic Diazepam Treatment in Middle-Aged Mice

**DOI:** 10.3389/fnagi.2021.777404

**Published:** 2021-11-26

**Authors:** Tomonori Furukawa, Yoshikazu Nikaido, Shuji Shimoyama, Nozomu Masuyama, Ayaka Notoya, Shinya Ueno

**Affiliations:** ^1^Department of Neurophysiology, Hirosaki University Graduate School of Medicine, Hirosaki, Japan; ^2^Department of Frailty Research and Prevention, Hirosaki University Graduate School of Medicine, Hirosaki, Japan; ^3^Department of Anesthesiology, Hirosaki University Graduate School of Medicine, Hirosaki, Japan; ^4^Research Center for Child Mental Development, Hirosaki University Graduate School of Medicine, Hirosaki, Japan

**Keywords:** benzodiazepine, chronic diazepam, middle-aged, cognitive function, exercise, LTP (long-term potentiation), spine

## Abstract

**Background:** Gamma-aminobutyric acid (GABA) type A receptors are positively allosterically modulated by benzodiazepine binding, leading to a potentiated response to GABA. Diazepam (DZP, a benzodiazepine) is widely prescribed for anxiety, epileptic discharge, and insomnia, and is also used as a muscle relaxant and anti-convulsant. However, some adverse effects – such as tolerance, dependence, withdrawal effects, and impairments in cognition and learning – are elicited by the long-term use of DZP. Clinical studies have reported that chronic DZP treatment increases the risk of dementia in older adults. Furthermore, several studies have reported that chronic DZP administration may affect neuronal activity in the hippocampus, dendritic spine structure, and cognitive performance. However, the effects of chronic DZP administration on cognitive function in aged mice is not yet completely understood.

**Methods:** A behavioral test, immunohistochemical analysis of neurogenic and apoptotic markers, dendritic spine density analysis, and long-term potentiation (LTP) assay of the hippocampal CA1 and CA3 were performed in both young (8 weeks old) and middle-aged (12 months old) mice to investigate the effects of chronic DZP administration on cognitive function. The chronic intraperitoneal administration of DZP was performed by implanting an osmotic minipump. To assess spatial learning and memory ability, the Morris water maze test was performed. Dendritic spines were visualized using Lucifer yellow injection into the soma of hippocampal neurons, and spine density was analyzed. Moreover, the effects of exercise on DZP-induced changes in spine density and LTP in the hippocampus were assessed.

**Results:** Learning performance was impaired by chronic DZP administration in middle-aged mice but not in young mice. LTP was attenuated by DZP administration in the CA1 of young mice and the CA3 of middle-aged mice. The spine density of hippocampal neurons was decreased by chronic DZP administration in the CA1 of both young and middle-aged mice as well as in the CA3 of middle-aged mice. Neither neurogenesis nor apoptosis in the hippocampus was affected by chronic DZP administration.

**Conclusion:** The results of this study suggest that the effects of chronic DZP are different between young and middle-aged mice. The chronic DZP-induced memory retrieval performance impairment in middle-aged mice can likely be attributed to decreased LTP and dendritic spine density in hippocampal neurons in the CA3. Notably, prophylactic exercise suppressed the adverse effects of chronic DZP on LTP and spine maintenance in middle-aged mice.

## Introduction

Benzodiazepines (BZDs) are positive allosteric modulators of gamma-aminobutyric acid type A receptors (GABAA-R) and potentiate GABAA-R activities, which results in the suppression of neural activity in the brain and spinal cord. Because this effect reduces anxiety, seizures, convulsions, muscle tone, and insomnia, BZDs have been widely prescribed as a medicine with high efficacy and low toxicity. However, the long-term use of BZDs is limited because of adverse effects such as tolerance, dependence, and withdrawal effects (Lader, 1991; Michelini et al., 1996; O’Brien, 2005). Additionally, it has been reported that long-term BZD administration induces cognitive decline in older adults, which provides evidence for another type of toxicity (Paterniti et al., 2002; Picton et al., 2018; Liu et al., 2020). Moreover, some clinical studies have reported that long-term BZD exposure increases the risk of incident Alzheimer’s disease in older adults (Yaffe and Boustani, 2014; Rosenberg, 2015). However, although guidelines generally recommend that BZDs are limited to short-term use, long-term use still often occurs.

The chronic administration of diazepam (DZP) or other BZDs elicits alterations at the molecular level, such as the downregulation of GABAA-R subunit gene expression (Pratt et al., 1998; Foitzick et al., 2020), internalization of GABAA-R (Ali and Olsen, 2001; Lorenz-Guertin et al., 2019), expression of glutamate receptor subunit genes (Allison and Pratt, 2003; Van Sickle et al., 2004), and expression of proteins associated with synaptic plasticity in the hippocampus (Monti et al., 2010, 2012). These findings are considered a probable mechanism for the tolerance, dependence, and withdrawal effects of BZDs. Additionally, structural changes involving decreased dendritic spine density have been described in the cortical pyramidal cells of chronic DZP-administered mice (Curto et al., 2016). Although the effects of BZDs have been extensively studied (del Cerro et al., 1992; McNamara et al., 1993; Joksimovic et al., 2013), the precise vulnerability of older adults to chronic BZD-induced cognitive decline is not yet fully understood.

In the aging process of mammals, defects in synaptic transmission and plasticity occur in different brain regions that are associated with cognitive function (Foster and Norris, 1997; Barnes, 2003; Disterhoft et al., 2004). The cognitive function involved in learning and memory is affected by several factors, such as neurogenesis, synaptic plasticity, and dendritic spine density (Drapeau et al., 2003; Bruel-Jungerman et al., 2005; Walker and Herskowitz, 2020); these factors decline with aging. For example, in aged rodents, the levels of neurogenesis are lower than in younger rodents (Kuhn et al., 1996; Kempermann et al., 1998; McDonald and Wojtowicz, 2005). In addition, aging-induced attenuation of hippocampal long-term potentiation (LTP) and dendritic spine density has also been observed (Nunzi et al., 1987; Arias-Cavieres et al., 2017). However, the effects of chronic BZD administration on such age-related changes remain unknown.

It has been reported that regular exercise prevents stress-induced neurotoxic effects such as dendritic retraction, spine loss in the hippocampus, and memory consolidation (Leem et al., 2019). In aged animals, exercise enhances brain health and cognitive performance (Larson et al., 2006; Ahlskog et al., 2011), and longitudinal studies have suggested that exercise enhances cognitive function in older adults (Kramer et al., 1999; Laurin et al., 2001; Barnes et al., 2003). In addition, it has been reported that exercise increases synaptic plasticity and LTP (van Praag et al., 1999), reverses dendritic spine loss (Toy et al., 2014), enhances hippocampal neurogenesis (van Praag et al., 2005), and upregulates brain-derived neurotrophic factor (BDNF) expression (Garza et al., 2004). These findings raise the possibility that exercise may have a preventive effect on chronic BZD-induced cognitive decline.

The present study was designed to investigate whether chronic DZP administration induces cognitive decline by modulating synaptic function and neurogenesis in the hippocampus. To examine the specific vulnerability of cognitive function in older adults to chronic DZP administration, both young and middle-aged mice were included. Furthermore, we investigated whether exercise has a protective effect in the hippocampus against the adverse consequences of chronic DZP administration.

## Materials and Methods

### Animals

Adult male C57BL/6J mice were used in this study. Eight-week-old mice were studied as the young group, while 12-month-old mice were used for the middle-aged group. Animals were group housed at 24 ± 2°C under a 12-h light/dark cycle (lights on at 8:00 a.m.), and food and water were available *ad libitum*. All experiments were performed in accordance with the guidelines for animal research issued by the Physiological Society of Japan and the Hirosaki University School of Medicine (approval number M12007), and all efforts were made to minimize the number of animals used and their suffering.

### Chronic Administration of Diazepam

Diazepam was chronically administered using ALZET osmotic pumps (Model 2004, 0.25 μL/h, 28-day duration; DURECT Corporation, Cupertino, CA, United States). Under isoflurane anesthesia, the ALZET osmotic pumps filled with either DZP or vehicle were intraperitoneally implanted. DZP was dissolved in 35% dimethyl sulfoxide (DMSO), 55% polyethylene glycol 400 (PEG 400), and 10% alcohol (70% volume/volume) (Vinkers et al., 2012). DZP concentrations were adjusted to deliver 5 mg/kg/day (Allison and Pratt, 2006).

### Morris Water Maze

Spatial memory was assessed using the Morris water maze (MWM) task in a circular pool (diameter: 95 cm, depth: 30 cm) filled with water at 23 ± 2°C (Bromley-Brits et al., 2011). During each trial, swimming trajectories of the mice were recorded using the CaptureStar video recording system (CleverSys Inc., Reston, VA, United States). An invisible escape platform (diameter, 10 cm) was placed 1 cm below the water surface. The visible platform task (60 s/trial × 5) was conducted using the flagged platform on Day 10. Mice that did not swim or reach the platform throughout this task were excluded for the following task. From days 11 to 14, the animals were then subjected to the hidden platform task (60 s/trial × 5/day × 4 days) to assess spatial acquisition. The latency to find the platform (escape latency, s), time spent swimming in the quadrant with the platform (target quadrant), and total swimming path length (m) were quantified in each trial using TopScan behavioral analysis software (CleverSys Inc.). On days 15 and 30, a probe test (60 s/test) was performed to estimate short- and long-term spatial memory formation. Escape latency and the percentage time spent in the target quadrant were analyzed. In this study, to define the duration of chronic DZP administration, the mice which was used in MWM were not used in other experiments.

### Histology and Immunohistochemistry

Neurogenesis and apoptosis in the hippocampus were studied histologically. The mice (*N* = 5/group) were transcardially perfused with 4% paraformaldehyde in 0.1 M phosphate buffer (PB), and the brains were then removed and postfixed in the same solution for 48 h. After postfixation, the brains were transferred to 20% sucrose in PB for 3 days. Tissue was cut coronally with a cryostat into 30 μm thick sections and rinsed in phosphate-buffered saline (PBS). To analyze neurogenesis, neurogenic differentiation factor 1 (NeuroD1; a marker of early neuronal differentiation) was stained immunohistochemically (Gao et al., 2009). Briefly, sections were rinsed in PBS-T buffer (0.1 M PBS, 1% Triton X-100) and incubated for 18 h at 4°C with antibodies against NeuroD1 (1:200, ab213725, monoclonal rabbit IgG; Abcam, Cambridge, United Kingdom). After rinsing in PBS, sections were incubated for 1 h with secondary antibodies against rabbit IgG (1:250, anti-rabbit biotinylated BA1000; Vector Laboratories, Burlingame, CA, United States). Visualization was performed using DyLight 488 streptavidin (1:500, SA-5488; Vector Laboratories). Primary and secondary antibodies were diluted in PBS containing 5% normal goat serum albumin and 0.1% Triton X-100. Apoptotic cell death was qualified by terminal deoxynucleotidyl transferase dUTP nick end labeling (TUNEL) staining using an *in situ* cell death detection kit (Sigma-Aldrich, San Diego, CA, United States) as per the manufacturer’s instructions. As a positive control, PBS-T-rinsed sections were incubated with DNase I (10 U/mL: Sigma-Aldrich) in PBS for 60 min at 37°C. After immunohistochemical or TUNEL staining, sections were stained with 4′,6-diamidino-2-phenylindole (DAPI), mounted on slides, coverslipped, and observed under a microscope (BZX-700; Keyence, Osaka, Japan). The numbers of stained cells in the hippocampus were counted using ImageJ software (National Institutes of Health, Bethesda, MD, United States) and presented as the density. The area in which positive cells were counted was also measured using ImageJ software.

### Electrophysiology

Mice (sedentary groups: *N* = 6 per group, exercise groups: *N* = 5 per group) were deeply anesthetized before their brains were rapidly removed and placed in cold (4°C), oxygenated, modified cutting artificial cerebrospinal fluid (ACSF) containing (in mM): 92 NaCl, 2.5 KCl, 1.2 NaH_2_PO_4_, 10.0 MgSO_4_, 2.0 CaCl_2_, 30.0 NaHCO_3_, 5.0 sodium ascorbate, 2.0 thiourea, 3.0 sodium pyruvate, 20.0 4-(2-hydroxyethyl)-1-piperazineethanesulfonic acid (HEPES), and 25.0 glucose. Transverse 350 μm sections that included the hippocampus were cut using a microslicer (Vibratome 3000; Vibratome, St Louis, MO, United States). Sections were incubated for 12 min in choline chloride-based recovery ACSF containing (in mM): 92 ChCl, 2.5 KCl, 1.2 NaH_2_PO_4_, 10.0 MgSO_4_, 2.0 CaCl_2_, 30.0 NaHCO_3_, 5.0 sodium ascorbate, 2.0 thiourea, 3.0 sodium pyruvate, 20.0 HEPES, and 25.0 glucose. After the incubation in recovery ACSF at 32°C, sections were maintained in recording ACSF containing (in mM): 126 NaCl, 2.5 KCl, 1.25 NaH_2_PO_4_, 1.2 MgSO_4_, 2.0 CaCl_2_, 26.0 NaHCO_3_, and 20.0 glucose. All solutions used in the experiment were gassed with 95% O_2_/5% CO_2_ prior to use. Next, individual sections were transferred to a chamber of the recording system (MED Probe; Alpha MED Scientific, Osaka, Japan), perfused with recording ACSF (2 mL/min), and maintained at 30°C. The field excitatory postsynaptic potentials (fEPSPs) of the CA1 or CA3 region of the hippocampus were recorded using a multi-electrode array system (MED64; Alpha MED Scientific). The fEPSPs were evoked (at 0.05 Hz) using stimulation strengths that were sufficient to elicit fEPSPs that were approximately 30% of the maximal fEPSP slope (10–90%). LTP was induced at 15 min of recording using a theta burst stimulation (TBS) paradigm. Offline analysis was performed using Mobius software (Alpha MED Scientific). LTP magnitude was measured as a percentage of the baseline fEPSP slope during the 15-min period just prior to TBS.

### Exercise Protocol

In the current study, forced running wheel (FRW) exercise was used. Before the chronic DZP administration, exercise was performed using a motor-driven running wheel (FWS-1505; Melquest Ltd., Toyama, Japan) for 20–50 min/day, 5 days/week for 12 weeks. As the FWS-1505 is programmable motorized wheel to control wheel speed, running time, and acceleration speed, the amount of exercise was equal for all exercise group mice. During the first week, the running speed and time were started at 2 m/min for 10 min, and then increased to 5 m/min for 10 min. The exercise was gradually increased for 5 weeks (week 2: 3 m/min for 10 min + increased to 5 m/min for 10 min + 5 m/min for 10 min, week 3: 3 m/min for 10 min + increased to 6 m/min for 10 min + 6 m/min for 10 min, week 4: 4 m/min for 10 min + increased to 7 m/min for 10 min + 7 m/min for 20 min, weeks 5–12: Initially 4 m/min, immediately increased to 7 m/min for 20 min + 7 m/min for 30 min).

### Spine Density Analysis

The protocol for spine density analysis was as previously described (Nishijima et al., 2014). Briefly, 4 weeks after the DZP administration, mice (*N* = 4 per group) were deeply anesthetized and intracardially perfused with 4% paraformaldehyde, and their brains were extracted and placed in PB. Coronal sections (250 μm) that included the hippocampus were prepared using a microslicer (VT-1000; Leica, Wetzlar, Germany). Neuronal spines were visualized using Lucifer yellow injection into pyramidal neurons of the CA1/3 by iontophoresis pump (BAB-600; Kation Scientific, Minneapolis, MN, United States) under a fluorescence microscope (BX51; Olympus, Tokyo, Japan). The sections were then mounted onto slides and coverslipped with Permount and SlowFade Gold Antifade Reagent (S36937; Invitrogen, Tokyo, Japan). Images of the Lucifer yellow-injected neurons were acquired using a laser confocal microscope (C1si; Nikon, Tokyo, Japan) with a 60 × oil-immersion lens. We then measured the density of spines on apical branches that were observed to be 100–200 μm from the cell body in the pyramidal cell layer of CA1 or CA3. Images (2–4 images/neuron) were acquired with zoom and were taken at 0.25 μm focal steps. Images were analyzed offline using NIS-Elements software (Nikon). The average number of spines per 20 μm of dendritic length was expressed as the spine density.

### Chemicals

The following drugs were used: DZP (FUJIFILM Wako Pure Chemical Industries, Osaka, Japan), PEG 400 (FUJIFILM Wako Pure Chemical), DMSO (Sigma-Aldrich), and Lucifer yellow (Sigma-Aldrich).

### Statistics

All values are presented as the mean ± SEM. Chi-squared tests were used to confirm the effects of DZP on MWM exclusion criteria. The unpaired *t*-test, one-way analysis of variance (ANOVA), or two-way ANOVA with repeated measures were performed to compare behavioral data. For the NeuroD1-positive cell counts, one-way ANOVA was performed. Simple comparisons of two groups (TUNEL-positive cell counts, spine density analysis, and LTP analysis) were performed using the Student’s *t*-test. For the LTP analysis, fEPSP slopes were constructed using the average of the last 15 min. Differences were considered statistically significant at *P* < 0.05.

## Results

### Effects of Chronic Diazepam Administration on Cognitive Function

**FIGURE 1:**
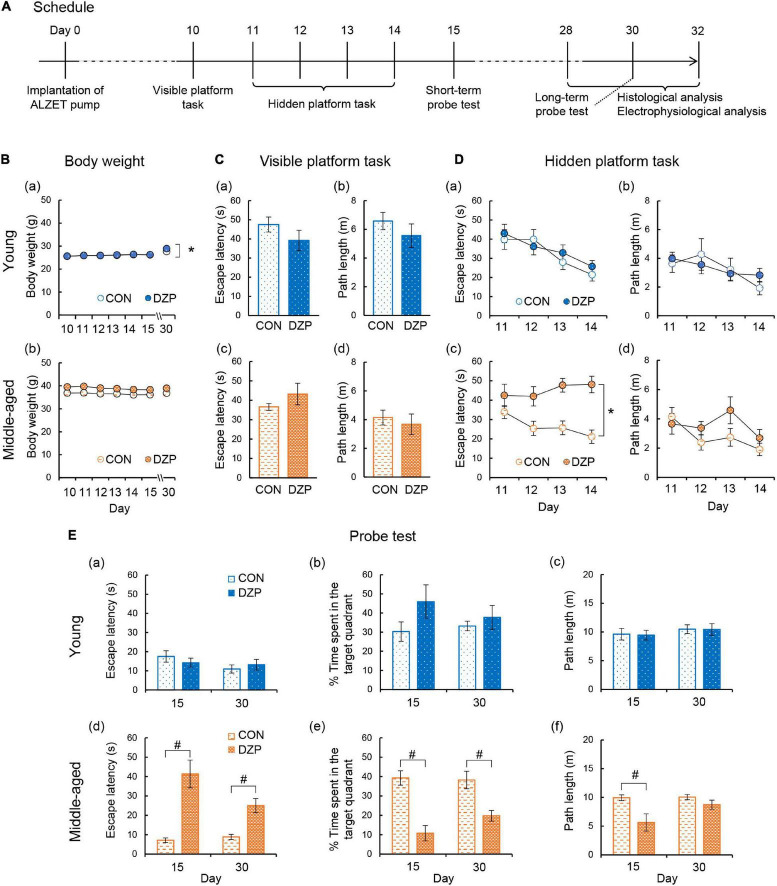
Effects of chronic diazepam administration on spatial memory performance. **(A)** The schedule of this research including Morris water maze, electrophysiological recordings, and histological analysis. **(B)** Changes in the body weight during the Morris water maze period. **(C–E)** Results of the Morris water maze test. **(C)** Escape latency and path length in the visible platform task of young and middle-aged mice. **(D)** Escape latency and path length in the hidden platform task from days 11 to 14. **(E)** Latency to the target quadrant, time spent in the target quadrant, and path length in the probe test on days 15 (short-term) and 30 (long-term). The number of mice that were analyzed in the tasks and probe test was as follows: young CON, *n* = 7; young DZP, *n* = 7; middle-aged CON, *n* = 8; middle-aged DZP, *n* = 7. Error bar: mean ± SEM. *P < 0.05, two-way analysis of variance with repeated measures. ^#^*P* < 0.05, *t*-test. CON, control group; DZP, diazepam group.

To evaluate the effects of chronic DZP treatment on spatial memory formation, the MWM was performed on four groups of mice: young control (CON, *n* = 11), young DZP-administered (DZP, *n* = 8), middle-aged CON (*n* = 14), and middle-aged DZP (*n* = 12). All mice were implanted with ALZET osmotic pumps filled with DZP or vehicle at day 0 (Figure 1A). After the osmotic pump implantation, young mice showed daily body weight gain during the MWM period independently of chronic DZP treatment [two-way ANOVA with repeated measures: “Treatment,” *F*(1,17) = 0.13, *P* > 0.05; “Day,” *F*(6,102) = 54.0, *P* < 0.05; “Treatment × Day,” *F*(6,102) = 3.71, *P* < 0.05; Figure 1Ba]. There were no significant effects of DZP on changes in body weight in middle-aged animals during the MWM period [*F*(1,24) = 2.87, *P* > 0.05; “Day,” *F*(6,144) = 1.02, *P* > 0.05; “Treatment × Day,” *F*(6,144) = 1.10, *P* > 0.05; Figure 1Bb]. Ten days after surgery, the visible platform task was performed. Four mice from the young CON group, one mouse from the young DZP group, six mice from the middle-aged CON group, and five mice from the middle-aged DZP group were excluded because they met the exclusion criteria for the visible platform task. Therefore, seven mice in the young CON group, seven in the young DZP group, eight in the middle-aged CON group, and seven in the middle-aged DZP group were included in the following behavioral analysis. The effects of DZP on the escape latency of young and middle-aged mice in the visible platform task were then examined. Chronic DZP treatment did not influence exclusion in either experimental set (young: χ^2^ = 1.36, *P* > 0.05; middle-aged: χ^2^ = 0.004, *P* > 0.05). There were no significant differences in escape latency and path length in the visible platform task [escape latency: young: *t*(12) = 1.36, *P* > 0.05; middle-aged: *t*(13) = 1.29, *P* > 0.05; path length: young: *t*(12) = 1.08, *P* > 0.05; middle-aged: *t*(13) = 0.59, *P* > 0.05; Figure 1C]. From days 11 to 14, the hidden platform task was performed, and the escape latencies and path lengths were plotted (Figure 1D). Young CON and DZP animals showed a stable learning performance in the hidden platform task (escape latency: “Treatment,” *F*(1,12) = 0.26, *P* > 0.05; “Day,” *F*(3,36) = 13.0, *P* < 0.05; “Treatment × Day,” *F*(3,36) = 0.81, *P* > 0.05; path length: “Treatment,” *F*(1,12) = 0.03, *P* > 0.05; “Day,” *F*(3,36) = 4.18, *P* < 0.05; “Treatment × Day,” *F*(3,36) = 1.91, *P* > 0.05; Figures 1Da,b]. The young mice also showed comparable spatial memory retrieval in the probe tests on days 15 and 30 (*P* > 0.05, CON vs. DZP; Figures 1Ea–c). In the middle-aged mice, escape latencies in the hidden platform task were significantly higher in the DZP group than in the CON group [“Treatment,” *F*(1,13) = 18.6, *P* < 0.05; “Day,” *F*(3,39) = 1.34, *P* > 0.05; “Treatment × Day,” *F*(3,39) = 4.39, *P* < 0.05; Figure 1Dc]. There were no significant differences in the path lengths between the CON and DZP groups [“Treatment,” *F*(1,12) = 0.03, *P* > 0.05; “Day,” *F*(3,36) = 4.18, *P* < 0.05; “Treatment × Day,” *F*(3,36) = 1.91, *P* > 0.05; Figure 1Dd]. In the subsequent probe tests on days 15 and 30, the middle-aged DZP mice showed impairment of spatial memory retrieval (*P* < 0.05, CON vs. DZP; Figures 1Ed,e). Their motor performance was reduced on day 15 (*P* > 0.05, CON vs. DZP; Figure 1Ef) but not day 30. These results indicate that chronic DZP treatment impairs spatial learning in middle-aged mice but not in young mice. Chronic DZP treatment did not influence motor performance in both age groups except for the short-term probe test. Additionally, to evaluate the effects of aging, behavioral performance was compared between the young and middle-aged groups. There were no significant differences in escape latency in the hidden platform task between the young CON and middle-aged CON groups [“Treatment,” *F*(1,13) = 2.26, *P* > 0.05; “Day,” *F*(3,39) = 10.5, *P* < 0.05; “Treatment × Day,” *F*(3,39) = 2.23, *P* > 0.05; Figures 1Da,c]. In the probe tests, there was a slight but significant difference in escape latency on day 15 only (*P* < 0.05), but mice in the CON group of both ages had equivalent memory retrieval performances (Figures 1Ea,b,d,e). Unlike the results from the CON groups, the middle-aged DZP group had significantly worse performances in both the hidden platform task [“Treatment,” *F*(1,12) = 5.18, *P* < 0.05; “Day,” *F*(3,36) = 1.43, *P* > 0.05; “Treatment × Day,” *F*(3,36) = 6.20, *P* < 0.05; Figures 1Da,c] and the probe test (*P* < 0.05, young DZP vs. middle-aged DZP; Figures 1Ea,b,d,e) compared with the young DZP group. These results suggest that the effects of chronic DZP treatment on behavioral performance in the MWM were stronger in middle-aged mice than in young mice.

### Effects of Chronic Diazepam Administration on Hippocampal Neurogenesis and Apoptosis

**FIGURE 2:**
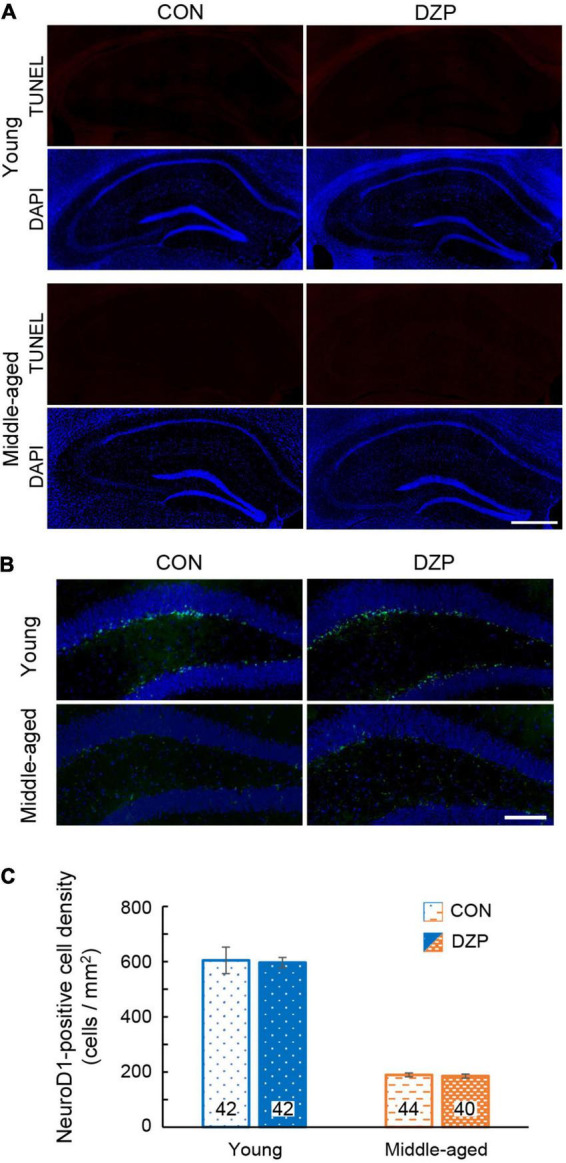
Effects of chronic diazepam administration on apoptosis and neurogenesis in the hippocampus. **(A)** Fluorescence microscopic image of TUNEL (red) and DAPI (blue) staining. Scale bar, 500 μm. **(B)** Immunofluorescence image of NeuroD1 (green) and DAPI (blue) staining in the hippocampus of young and middle-aged mice. Scale bar, 50 μm. **(C)** Density of NeuroD1-positive cells in the dentate gyrus. The number in bar graph indicate the number of sections (*N* = 5 animal, 7–9 sections/animal). Error bar: mean ± SEM. CON, control group; DAPI, 4′,6-diamidino-2-phenylindole; DZP, diazepam group; NeuroD1, neurogenic differentiation factor 1; TUNEL, terminal deoxynucleotidyl transferase dUTP nick end labeling.

Neurogenesis in the adult dentate gyrus of the hippocampus occurs constitutively throughout postnatal life, and the rate of neurogenesis alters cognitive functions (Ansorg et al., 2012). In addition, apoptosis in the hippocampus is associated with cognitive deficits (Sima and Li, 2005). To examine the effects of chronic DZP administration on neurogenesis and apoptosis, TUNEL and NeuroD1 staining were performed in the hippocampus. There were very few TUNEL-positive cells in the hippocampus (≤5 cells/section) among all groups (Figure 2A; young CON: *n* = 40, young DZP: *n* = 41, middle-aged CON: *n* = 41, middle-aged DZP: *n* = 39 sections). NeuroD1-positive cells were detected in the dentate gyrus in all groups (Figures 2B,C; young CON: *n* = 42, young DZP: *n* = 42, middle-aged CON: *n* = 44, middle-aged DZP: *n* = 40 sections). There was a significant reduction in the number of NeuroD1-positive cells with age (Figure 2C and Supplementary Figure 1Aa; young vs. middle-aged: *P* < 0.01, one-way ANOVA, Tukey’s test) but no significant differences between the CON and DZP groups (Figure 2C; young CON vs. young DZP: *P* = 0.97; middle-aged CON vs. middle-aged DZP: *P* = 1.00; one-way ANOVA, Tukey’s test). These results suggest that neurogenesis is reduced with aging, and that the regulation of apoptosis and neurogenesis in the hippocampus is not affected by chronic DZP administration.

### Hippocampal Long-Term Potentiation in Chronic Diazepam-Administered Mice

**FIGURE 3:**
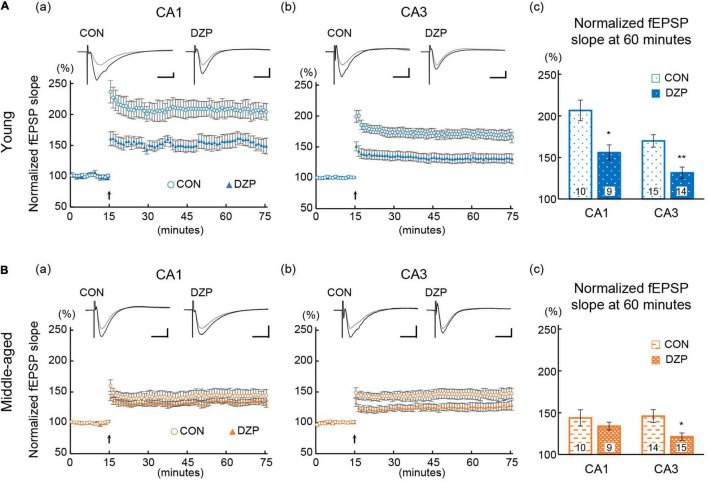
Effects of chronic diazepam administration on long-term potentiation in the hippocampal CA1 and CA3 regions of young **(A)** and middle-aged **(B)** mice. Long-term potentiation was induced by TBS at 15 min of recording. The typical fEPSP responses (upper **a,b**) from 5 min before (gray) and 45 min after (black) the TBS are superimposed. The peak response of 5 min before TBS was normalized to 100%. Scale bar indicates 0.3 mV/10 ms in **(A)** and 0.2 mV/10 ms in **(B)**. The plot shows normalized fEPSP slopes every 1 min. The average normalized fEPSP slopes from the last 15 min were used to compare the magnitude of long-term potentiation between CON and DZP. The number in bar graph indicate the number of recorded slices (*N* = 6 animal, 1–3 slices/animal). Error bar: mean ± SEM. *P < 0.05, Student’s *t*-test; **P < 0.01, Student’s *t*-test; vs. CON. CON, control group; DZP, diazepam group; fEPSP, field excitatory postsynaptic potentials.

Many studies have suggested that synaptic plasticity of the hippocampus is related to spatial memory abilities (Morris et al., 1982; Baez et al., 2018). LTP was induced in the hippocampal CA1 and CA3 to evaluate synaptic plasticity in chronic DZP-administered mice. The magnitude of LTP was assessed at 45 min after TBS. Hippocampal LTP was impaired by chronic DZP administration in both the CA1 and CA3 in young mice [CA1: CON (*n* = 10) vs. DZP (*n* = 9), *P* = 0.01; CA3: CON (*n* = 15) vs. DZP *(n* = 14), *P* < 0.01; Figure 3A]. In middle-aged mice, a significant reduction of LTP was also observed in the CA3 of the DZP-administered group [CA1: CON (*n* = 10) vs. DZP (*n* = 9), *P* = 0.70; CA3: CON (*n* = 14) vs. DZP (*n* = 15), *P* = 0.04; Figure 3B]. Additionally, the LTP magnitude was significantly reduced by aging in the CA1 and CA3 (Supplementary Figure 1Ab: CA1: young vs. middle-aged CON, *P* < 0.01; CA3: young vs. middle-aged CON, *P* < 0.05). These results indicate that LTP, which is impaired in aging, is further attenuated by chronic DZP administration in the CA3 of middle-aged mice.

### Spine Density of Hippocampal Neurons in Chronic Diazepam-Administered Mice

**FIGURE 4:**
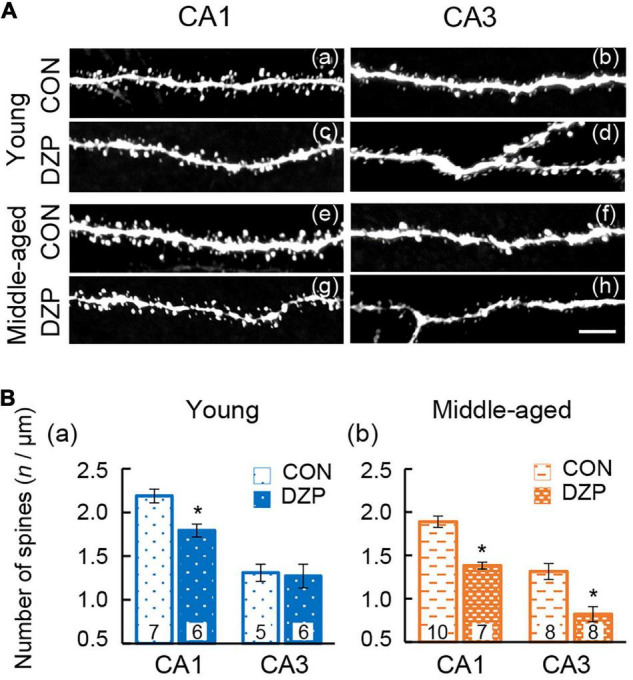
Effects of chronic diazepam administration on the spine density of hippocampal neurons. **(A)** Representative images of visualized spines in the CA1 and CA3 regions of young and middle-aged mice. **(B)** Spines were counted and the results are summarized in bar graphs. The number in bar graph indicate the number of analyzed neurons (*N* = 4 animals, 1–3 neurons/animal). Scale bar, 5 μm. Error bar: mean ± SEM. *P < 0.01, *t*-test, vs. CON. CON, control group; DZP, diazepam group.

There is wide agreement that the integrity of the hippocampal neural network contributes to spatial memory behavior. To investigate the effects of DZP on synaptic connections in the hippocampal neural network, dendritic spine density was analyzed in both the CA1 and CA3 of the hippocampus (Figure 4A). Pyramidal neuron spines in the hippocampus were visualized using intracellular injections of Lucifer yellow into the soma in fixed sections, and spine density was then examined. In young mice, chronic DZP administration reduced spine density in the CA1 but not the CA3 [CA1: CON (*n* = 7) vs. DZP (*n* = 6), *P* < 0.01; CA3: CON (*n* = 5) vs. DZP (*n* = 6), *P* = 0.85; Figure 4Ba]. In middle-aged mice, spine density was reduced by DZP in both the CA1 and CA3 [CA1: CON (*n* = 10) vs. DZP (*n* = 7), *P* < 0.01; CA3: CON (*n* = 8) vs. DZP (*n* = 8), *P* < 0.01; Figure 4Bb]. In the CA1 of the hippocampus, there was also a significant reduction in spine density with aging (Supplementary Figure 1Ac: *P* < 0.01). These results suggest that dendritic spine maintenance is impaired by chronic DZP administration, especially in middle-aged mice.

### Exercise Improves Diazepam-Induced Impairments in Spine Density and Long-Term Potentiation in Hippocampal Neurons

**FIGURE 5:**
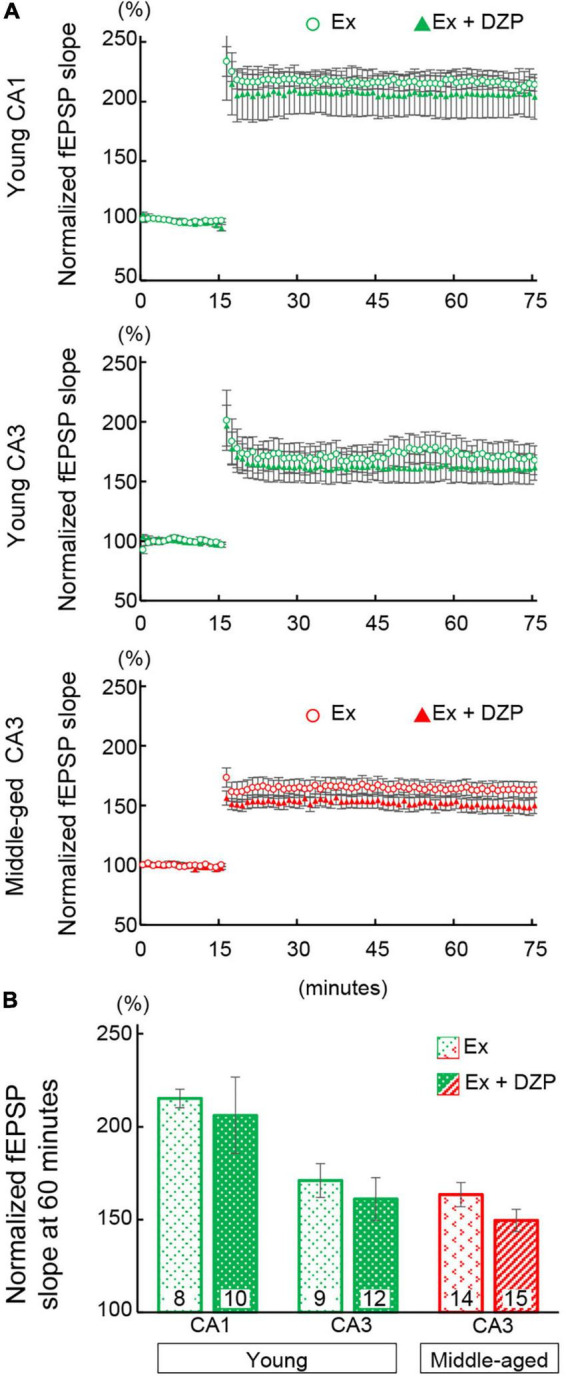
Effects of exercise on hippocampal long-term potentiation in chronic diazepam- or vehicle-administered young and middle-aged mice. **(A)** Averages of every 1 min of normalized fEPSP slopes were plotted. Theta burst stimulation was delivered at 15 min of recording to induce long-term potentiation. **(B)** To compare the magnitudes of long-term potentiation, the normalized fEPSP slopes of 60- to 75-min recordings were averaged and are summarized as a bar graph. The number in bar graph indicate the number of recorded slices (*N* = 5 animal, 1–3 slices/animal). Error bar: mean ± SEM. Ex, exercise group; Ex + DZP, exercise + diazepam group; fEPSP, field excitatory postsynaptic potentials.

**FIGURE 6:**
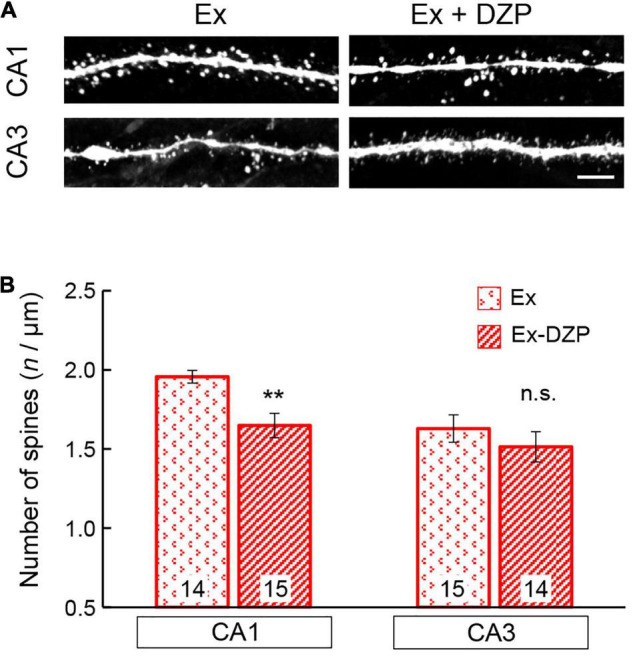
Effects of exercise on the spine density of hippocampal neurons in middle-aged mice. **(A)** Representative images of visualized spines in the CA1 and CA3 regions of middle-aged mice. **(B)** Spine density was measured and is summarized in a bar graph. The number in bar graph indicate the number of analyzed neurons (*N* = 4 animals, 3–4 neurons/animal). Scale bar, 5 μm. Error bar: mean ± SEM. **P < 0.01, *t*-test, vs. Ex group; n.s., not significant. Ex, exercise group; Ex + DZP, exercise + diazepam group.

The facilitation or improvement of hippocampal LTP has been attempted using various approaches, including exercise. In the present study, we examined whether FRW exercise was able to attenuate the chronic DZP-induced effects on the hippocampus. After 12 weeks of FRW exercise, hippocampal LTP and spine density were investigated in chronic DZP-administered mice. In both the CA1 and CA3 of young mice and the CA3 of middle-aged mice (all of which were significantly affected by DZP administration in sedentary mice; Figure 3), there was no significant reduction in LTP magnitude with chronic DZP administration [young CA1: exercise (Ex) (*n* = 8) vs. Ex + DZP (*n* = 10), *P* = 0.69; young CA3: Ex (*n* = 9) vs. Ex + DZP (*n* = 12), *P* = 0.58; middle-aged CA3: Ex (*n* = 14) vs. Ex + DZP (*n* = 15), *P* = 0.12; Figures 5A,B]. Likewise, in the analysis of dendritic spine density in middle-aged mice, a significant difference remained in the CA1 but not in the CA3 [CA1: Ex (*n* = 14) vs. Ex + DZP (*n* = 15), *P* < 0.01; CA3: Ex (*n* = 15) vs. Ex + DZP (*n* = 14), *P* = 0.20; Figures 6A,B]. Furthermore, when comparing between the DZP and Ex + DZP groups, dendritic spine density in the CA1 of the exercise group was significantly higher than that of the sedentary group (Supplementary Figure 1B). These results indicate that the chronic DZP-induced reductions in dendritic spine density and LTP magnitude are preventable by FRW exercise before the DZP administration.

## Discussion

In the present study, we investigated the effects of chronic DZP administration on hippocampal function in both young and middle-aged mice. In the MWM test, cognitive performance was impaired by chronic DZP administration in middle-aged mice but not in young mice. This result suggests that the cognitive performance of middle-aged mice is more vulnerable to chronic DZP administration than that of young mice. Partially consistent with the results of the behavioral test, both hippocampal LTP in the CA3 and spine density were reduced in middle-aged mice by chronic DZP, although there was no modulation of neurogenesis or apoptosis. Consistent with this finding, previous studies have reported that DZP administration does not alter neurogenesis or apoptosis (Sarnowska et al., 2009; Wu and Castren, 2009; Nochi et al., 2013). In chronic DZP-administered young mice, although a reduction in cognitive performance was not observed in the MWM test, there was reduced hippocampal LTP and spine density in these animals. These results suggest that, regardless of behavioral cognitive performance, chronic DZP administration induces impairments in synaptic plasticity and spine maintenance in hippocampal neurons. In contrast, NeuroD1- and TUNEL-positive signals were not affected by chronic DZP administration in young or middle-aged mice. Together, our results suggest that chronic DZP-induced cognitive decline in middle-aged mice is not caused by changes in neurogenesis or apoptosis. In addition to the effects of DZP, there were aging-related effects on hippocampal neurons. Both LTP magnitude and neurogenesis were significantly lower in middle-aged mice than in young mice (Supplementary Figures 1Aa,b). In DZP administered mice, LTP magnitude of young mice was higher than that of aged mice. Especially in CA1, LTP magnitude of DZP treated young mice was higher than middle-aged control mice. Taking these findings into account, the vulnerability of middle-aged mice to chronic DZP administration may be attributed to aging. The effects of aging and chronic DZP administration on hippocampal neurons were similar, and the cognitive impairment that was observed in middle-aged mice in the MWM test might be induced by a synergistic effect of aging and chronic DZP administration. This synergistic effect could explain the fact that there was contrasting data between MWM and LTP with regards to both young and aged mice.

The effects of chronic DZP administration on spine loss and LTP suppression were observed in the hippocampal neurons of both young and middle-aged mice. We have previously reported that chronic DZP administration increases lipocalin 2 expression (Furukawa et al., 2017), and another recent study revealed that chronic BZD administration to aged mice upregulates amyloid beta 42 expression and downregulates neprilysin expression (Jung et al., 2020). The expression of such molecules at normal levels is essential for neural excitability, spine maintenance, and synaptic plasticity (Huang et al., 2006; Lauren et al., 2009; Mucha et al., 2011). Thus, the chronic DZP-induced adverse effects of the present study might be associated with these molecules. Additionally, the chronic DZP-induced adverse effects might trigger activation of those molecules by modulating GABAA-R activity. If so, treatment of flumazenil, a competitive benzodiazepine receptor antagonist, would prevent or attenuate the chronic DZP effects. It was reported that there are both beneficial and adverse effect of flumazenil treatment for cognitive function (Neave et al., 2000; Colas et al., 2017). Further research will be needed to clarify the effect of flumazenil on cognitive impairment induced by chronic DZP.

Meanwhile, numerous studies suggested correlation between spine density and LTP in hippocampus (Zhai et al., 2020). However, our results were not completely consistent between LTP and spine density. In young mice, although LTP of CA3 was significantly reduced, spine density of CA3 were not decreased. Occasionally, it was reported that LTP was impaired without affecting spine density in the hippocampus of drebrin A knock out mice (Kojima et al., 2016). Additionally, the LTP of middle-aged mice were significantly or tend to reduce by chronic DZP, however the magnitude of decrease in LTP was smaller than that of young mice, even though magnitudes of decrease by DZP in spine density were not so remarkable. Our study could not explain these unexpected results, and the maintenance mechanism of spine density in aged animal was not fully elucidated. It was assumed that responses and function of molecules associated with LTP induction and spine maintenance might be modified by aging and different in regions. Further investigation is necessary to clarify the underlying molecular mechanisms.

Age-related declines in cognitive function have been explored in numerous studies. Several reports have revealed that cognitive function in mice aged 18 months or older is lower than that in young mice (van Praag et al., 2005; Oliveira et al., 2012). However, our data revealed that the cognitive performance of middle-aged control mice in the MWM test was nearly identical to that of young control mice. This discrepancy might be attributed to the differences in mouse ages. The 12-month-old mice used in the present study might not have been sufficiently old enough to exhibit age-related cognitive decline in this behavioral test (Tsai et al., 2018). However, we did observe age-related reductions in hippocampal LTP and neurogenesis in these mice. Together, these findings suggest that middle-aged mice might be vulnerable to chronic DZP administration even though they do not show cognitive impairment in behavioral tests.

The preventive effects of regular physical exercise against cognitive deterioration and dementia risk have been reported (Paillard, 2015). In the current study, we examined whether the adverse effects of chronic DZP administration were prevented by physical exercise. The chronic DZP-induced reductions in LTP magnitude and spine density were attenuated by FRW exercise for 12 weeks. These results suggest that physical exercise may be a potential treatment to protect against cognitive decline caused by chronic DZP. However, our data were unable to elucidate the molecular mechanisms of these exercise-induced beneficial effects on hippocampal neurons. Previous studies have demonstrated that exercise increases BDNF and tropomyosin receptor kinase B mRNA expression levels in the brains of rodents (Gomez-Pinilla et al., 2002; Garza et al., 2004; Jin et al., 2017). BDNF is a protein that promotes neural survival and the differentiation of new neurons and synapses. BDNF is thought to be a major contributor toward enhancing memory and increasing spine density (Leem et al., 2019). In addition to BDNF, the expression of other growth factors – such as fibroblast growth factor (Gomez-Pinilla et al., 1997, 1998), insulin-like growth factor I (Stein et al., 2018), and nerve growth factor (Chae and Kim, 2009) – are elevated by exercise. These factors play an important role in neural function in the adult brain (van Praag, 2008). It is therefore likely that the expression of growth factors was upregulated by exercise even in the hippocampus of chronic DZP-administered mice in the present study, thus leading to the observed beneficial effects in hippocampal neurons. Furthermore, a recent study identified exercise-responsive peripheral molecules that are associated with protective effects against cognitive decline (McGee and Hargreaves, 2020). For example, the peripheral overexpression of fibronectin type III domain-containing protein 5 rescues synaptic plasticity and memory in Alzheimer’s disease model mice (Lourenco et al., 2019). In addition, glycosylphosphatidylinositol-specific phospholipase D1 in plasma is increased by exercise, and is related to improved cognitive function in middle-aged mice (Horowitz et al., 2020). Such molecules may have been functional even in our model mice, but further research is needed to reveal the detailed mechanisms of exercise-induced protective effects in chronic DZP-administered mice.

Exercise enhances cognitive function and prevents age-related memory decline (Gomes da Silva et al., 2012). However, in the current study, although we observed age-related reductions in dendritic spine density and impairments in LTP, FRW exercise did not improve the effects of aging in the control group. It is possible that the intensity of FRW exercise that was used in this study was not sufficient to improve these effects of aging. Additionally, our mice may not have been old enough to be able to observe any FRW exercise-induced improvements of age-related effects in the hippocampus.

It is well known that sedation was produced by BZDs. The injection of BZD induces sedative effects after the acute injection. However, tolerance is induced by chronic BZD administrations, and sedation is progressively decreased through 7 days (Griffiths et al., 1992). In this study, MWM experiments were started at 10 days after the osmotic pump implantation. On the day performing visible platform task, body weight and path length, indicating motor performance, were not significantly different between control and DZP groups of both young and middle-aged mice. These results indicated that sedative effects of DZP have disappeared by day 10 in the MWM experiment. Meanwhile, there was a possibility that DZP-induced sedation and home cage behaviors might be affected by aging and/or exercise. However, as our data were confined to chronic DZP effects, the effects of aging and exercise on DZP-induced sedation and home cage behaviors could not be defined. In future, further studies about home cage behaviors and physiological parameters of exercised mice before and following DZP administration would reveal detail mechanism and availability of exercise against to the effect of aging and DZP administration.

In conclusion, we demonstrated that synaptic plasticity and dendritic spine density were reduced in the hippocampus by chronic DZP administration. In middle-aged mice, although no behavioral signs of cognitive decline were observed, there were several age-related alterations in the hippocampus. These alterations were likely contributing factors to the vulnerability of cognitive function to chronic DZP administration in the middle-aged mice. Furthermore, our findings provide evidence that FRW exercise may reduce the effects of chronic DZP administration on the hippocampus; however, further research is needed to identify the underlying molecular mechanisms.

## Data Availability Statement

The original contributions presented in the study are included in the article/Supplementary Material, further inquiries can be directed to the corresponding author.

## Ethics Statement

The animal study was reviewed and approved by the Hirosaki University School of Medicine.

## Author Contributions

TF and SU designed the study. YN performed the behavioral experiments. TF, NM, and AN performed the electrophysiological, imaging, and histochemical experiments. TF, YN, SS, NM, and AN analyzed the data. TF and YN wrote the manuscript with contributions from SU. All authors read and approved the final manuscript.

## Conflict of Interest

The authors declare that the research was conducted in the absence of any commercial or financial relationships that could be construed as a potential conflict of interest.

## Publisher’s Note

All claims expressed in this article are solely those of the authors and do not necessarily represent those of their affiliated organizations, or those of the publisher, the editors and the reviewers. Any product that may be evaluated in this article, or claim that may be made by its manufacturer, is not guaranteed or endorsed by the publisher.
