# Supplementary material for: Impaired Cognitive Function and Hippocampal Changes Following Chronic Diazepam Treatment in Middle-Aged Mice
Source: Front Aging Neurosci. 2021 Nov 26;13:777404. doi: 10.3389/fnagi.2021.777404 (PMC8664496; doi:10.3389/fnagi.2021.777404)
Supplement: Supplementary file 1 [file Presentation_1.pptx]

## Slide 1
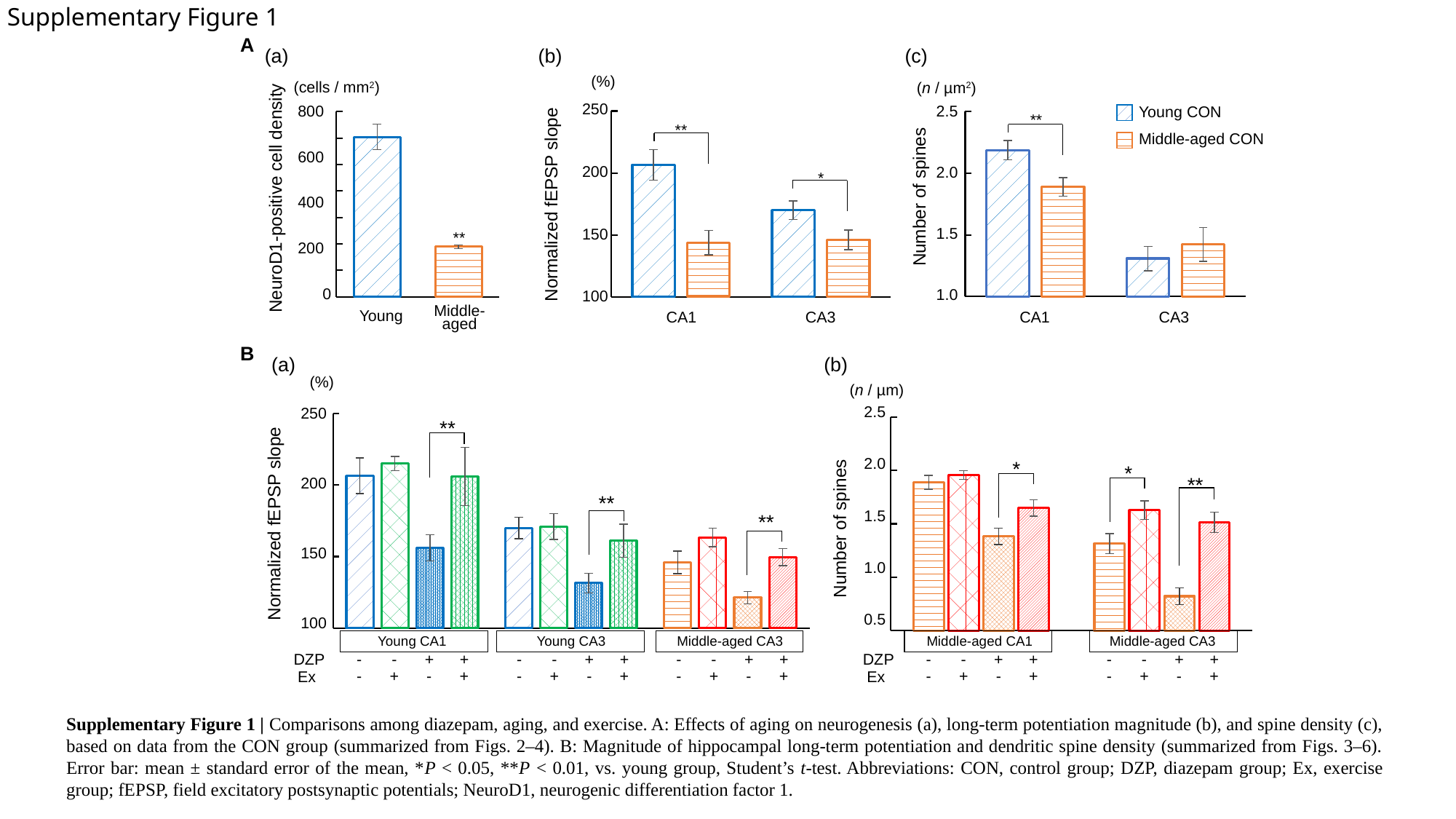

Supplementary Figure 1
A
(a)
(b)
(c)
(%)
250
200
150
100
### Chart
| Category | Y-CON | A-CON |
|---|---|---|
| CA1 | 2.1875 | 1.8888888888888888 |
| CA3 | 1.309090909090909 | 1.4224999999999999 |(cells / mm2)
(n / µm2)
### Chart
| Category | CON |
|---|---|
| Young | 604.5160839187046 |
| Aged | 189.4080679008115 |
### Chart
| Category | | |
|---|---|---|
| CA1 | 206.52244610540228 | 143.7709195755448 |
| CA3 | 169.9913619478909 | 145.95470934915096 |800
600
400
200
0
2.5
2.0
1.5
1.0
Young CON
**
**
Middle-aged CON
*
NeuroD1-positive cell density
Number of spines
Normalized fEPSP slope
**
Young
Middle-aged
CA1
CA3
CA1
CA3
B
(a)
(b)
(%)
250
200
150
100
(n / µm)
### Chart
| Category | CON-SED | CON-EX | DZP-SED | DZP-Ex |
|---|---|---|---|---|
| Y-CA1 | 206.52244610540228 | 215.12115232293357 | 156.0301226176411 | 206.10822767597702 |
| Y-CA3 | 169.9913619478909 | 171.02364969809614 | 131.48858009193182 | 161.0386024125001 |
| A-CA3 | 145.95470934915096 | 163.42844239470008 | 121.24914534544158 | 149.5327113538692 |2.5
2.0
1.5
1.0
0.5
### Chart
| Category | Con | DZP | Ex-Con | Ex-DZP |
|---|---|---|---|---|
| | 1.8888888888888888 | 1.95625 | 1.3821428571428571 | 1.6486842105263158 |
| | 1.3142857142857143 | 1.6290697674418606 | 0.821875 | 1.5137254901960784 |**
*
*
**
**
**
Normalized fEPSP slope
Number of spines
Middle-aged CA1
Middle-aged CA3
Young CA1
Young CA3
Middle-aged CA3
DZP
-
-
+
+
-
-
+
+
-
-
+
+
DZP
-
-
+
+
-
-
+
+
-
+
-
+
-
+
-
+
-
+
-
+
-
+
-
+
-
+
-
+
Ex
Ex
Supplementary Figure 1 | Comparisons among diazepam, aging, and exercise. A: Effects of aging on neurogenesis (a), long-term potentiation magnitude (b), and spine density (c), based on data from the CON group (summarized from Figs. 2–4). B: Magnitude of hippocampal long-term potentiation and dendritic spine density (summarized from Figs. 3–6). Error bar: mean ± standard error of the mean, *P < 0.05, **P < 0.01, vs. young group, Student’s t-test. Abbreviations: CON, control group; DZP, diazepam group; Ex, exercise group; fEPSP, field excitatory postsynaptic potentials; NeuroD1, neurogenic differentiation factor 1.
